# Supplementary material for: Outcomes of metabolic syndrome and anxiety levels in light and heavy smokers
Source: PeerJ. 2025 Mar 5;13:e19069. doi: 10.7717/peerj.19069 (PMC11890032; doi:10.7717/peerj.19069)
Supplement: Supplemental Information 1 [file peerj-13-19069-s001.docx]

**CODEBOOK OF DATASET**

A2= Age

B2= Gender (1= Male, 2=Female)

C2= Chronic Disease (1= Exist, 2= None)

D2= First Cigarete (minutes)

E2= Fargestrom Points

F2-Y2= State Anxiety Points

Z2-AS2= Trait Anxiety Points

AT2= Fasting Glucose

AU2= Hemoglobin

AV2=Hematocrite

AW2= Trygliseride

AX2= Total Alcohol

AV2= Ldl

HDL

Insuline

HgbA1C

Ast

Alt

Height (cm)

Weight (kg)

Systolic BP

Diastolic BP

Height (m)

Bmı

Bmı Groups (1= Underweight, 2= Normal, 3= Overweight, 4= Obesity)

Durumluk= State Anxiety Total Points

Sürekli= Trait Anxiety Total Points

Filter_$= (1= Selected: Systolic>=130 | Diastolic BP>=85)

AKŞLEVELS= Fasting Blood Glucose

Kaçyıliçici= Smoking years

Paket yıl= Pack Years

Ağır.Hafif.Orta.İçicilik= Smoking Status by Pack Years (1=Light smoker, 2= Moderate, 3=Severe)

PY.10.20.30= Pack Years Quartiles (1= Up to 10 years, 2= 10<PY<20, 3= 20<PY<30, 4= 30<PY)

High Trigliseride= (0= Normal, 1= High TG)

Low Hdl= (0= Normal, 1= Low Hdl)

Blood Pressure= (0= Normal, 1= High BP)

Fasting Glucose= (0= Normal, 1= High Glucose Level)

Bozulmuş Açlık Glikozu= İmpairement Fastin Glucose (0= Normal, 1= IFG, 2=DM)

MedainaGöreİçicilik= Smoking status by median Pack years (1=Light, 2= Heavy)

Durumluk.SEVİYELERİ= State Anxiety Status (1= Normal, 2= Moderate, 3= Severe)

Sürekli.SEVİYELERİ= Traite Anxiety Status (1= Normal, 2= Moderate, 3= Severe)

Homa

Durumluk.Normal.Moderate= State Anxiety Status (1=Normal and Moderate, 2= Severe)

Sürekli.Normal.Moderate= Trait Anxiety Status (1=Normal and Moderate, 2= Severe)
