# Supplementary material for: Outcomes of metabolic syndrome and anxiety levels in light and heavy smokers
Source: PeerJ. 2025 Mar 5;13:e19069. doi: 10.7717/peerj.19069 (PMC11890032; doi:10.7717/peerj.19069)
Supplement: Supplemental Information 2 [file peerj-13-19069-s002.docx]

STROBE Statement—checklist of items that should be included in reports of observational studies

|  | Item No. | Recommendation | Page  No. | Relevant text from manuscript |
| --- | --- | --- | --- | --- |
| **Title and abstract** | 1 | (*a*) Indicate the study’s design with a commonly used term in the title or the abstract | 1 | This cross-sectional study was conducted at a smoking cessation clinic in Turkey |
|  |  | (*b*) Provide in the abstract an informative and balanced summary of what was done and what was found | 1 | Design: This cross-sectional study was conducted at a smoking cessation clinic in Turkey, enrolling individuals who visited the clinic in 2022. The Fagerstrom Test for Nicotine Dependence and the State-Trait Anxiety Inventory were utilized as assessment tools, while metabolic syndrome parameters (body mass index, hypertension, hyperglycemia, dyslipidemia) were evaluated. Smoking status was classified based on pack-years.  Results: The study revealed a dose-dependent relationship between smoking status and essential metabolic factors such as systolic blood pressure (SBP), diastolic blood pressure (DBP), HgbA1C, and low-density lipoprotein (LDL). Notably, triglyceride (TG) levels exhibited a significant increase, particularly at 25 pack years. While anxiety levels did not exhibit a significant correlation with smoking status, they demonstrated an upward trend with increasing SBP and DBP values. |
| Introduction | | | |  |
| Background/rationale | 2 | Explain the scientific background and rationale for the investigation being reported | 2 | Smoking constitutes a chronic addiction to nicotine, acknowledged as a significant risk factor for a spectrum of diseases and disabilities impacting both physical and mental well-being [1]. Tobacco smoking is one of the biggest public health risks in the general population. According to the World Health Organization (WHO), over 8 million people die annually due to tobacco-related causes, with over 7 million directly attributable to tobacco use, and 1.2 million fatalities resulting from health complications associated with passive smoking [2]. If current smoking trends persist, projections suggest that more over 8 million individuals will succumb annually to tobacco-related illnesses by the year 2030 [3]. Notably, individuals with psychiatric disorders exhibit a heightened prevalence of nicotine addiction, as nicotine's psychoactive properties often temporarily alleviate psychiatric symptoms to some extent [4]. Despite mounting evidence of its detrimental effects, smoking remains ingrained in the belief system of many, who perceive it as a stress reliever and anxiety-reducer [5]. Particularly noteworthy is the disproportionately elevated smoking prevalence among individuals with psychiatric conditions such as schizophrenia, anxiety disorders, eating disorders, and attention deficit hyperactivity disorder [6].  A literature review highlights a significant failure rate in smoking cessation efforts. Studies examining smoking cessation programs underscore a concerning trend of high failure rates, largely attributed to elevated levels of depression, anxiety, and stress, coupled with a general reluctance to adopt behavioral changes [7, 8]. Anxiety, given its role as a potential motivator for smoking initiation, emerges as a crucial focus area warranting investigation [9]. Notably, smokers grappling with heightened anxiety levels often experience poorer outcomes in cessation efforts, marked by intensified withdrawal symptoms and a heightened urge to smoke [10, 11]. Moreover, apprehensions surrounding nicotine withdrawal symptoms and heightened state anxiety further compound the challenges of quitting smoking for individuals with elevated anxiety levels [12]. On a positive note, emerging evidence suggests smoking cessation can complement treatment strategies for mental disorders, particularly anxiety disorders, yielding favorable effects on mental health outcomes [13, 14].  Metabolic syndrome, characterized by a cluster of cardiovascular risk factors, including abdominal obesity, hypertension, hyperglycemia, and dyslipidemia, represents a burgeoning public health concern with implications for diabetes and cardiovascular morbidity; approximately one-quarter of the global adult population is documented to have metabolic syndrome [15, 16]. Lifestyle shifts toward sedentary behaviors and high-calorie diets have fueled the escalating prevalence of metabolic syndrome, posing a formidable threat to recent strides in health advancement [17]. [18] |
| Objectives | 3 | State specific objectives, including any prespecified hypotheses | 2-3 | The association between anxiety and smoking behavior often leads individuals to smoke more, thereby increasing their exposure to the adverse effects of smoking and the risk of developing metabolic syndrome. This underscores the importance of conducting comprehensive evaluations and providing integrated services for individuals seeking smoking cessation assistance at outpatient clinics. There is a need in the literature in terms of studies which concurrently investigate smoke consumption, anxiety, and metabolic syndrome. Additionally, studies on the effect of nicotine addiction on metabolic syndrome have yielded conflicting results, highlighting the need for further research. Therefore, this study aimed to evaluate the effect of smoking status, nicotine dependence on metabolic syndrome parameters and simultaneously examining anxiety levels in smokers. By examining these factors concurrently, we aim to contribute to a better understanding of the complex interplay between smoking behavior, psychological factors, and metabolic health, thereby informing more effective strategies for smoking cessation interventions and metabolic syndrome management. |
| Methods | | | |  |
| Study design | 4 | Present key elements of study design early in the paper | 3 | This cross-sectional investigation was carried out at a smoking cessation outpatient facility within the provincial health directorate of Adana, located in Southern Turkey, targeting individuals expressing a willingness to cease smoking. |
| Setting | 5 | Describe the setting, locations, and relevant dates, including periods of recruitment, exposure, follow-up, and data collection | 3 | Sampling  This cross-sectional investigation was carried out at a smoking cessation outpatient facility within the provincial health directorate of Adana, located in Southern Turkey, targeting individuals expressing a willingness to cease smoking. Enrolment comprised all smokers seeking assistance at the smoking cessation outpatient clinic, with inclusion spanning all visitors to the facility throughout 2022 during their initial consultation. Exclusion criteria were predetermined confounding factors, including a current body mass index exceeding 30, presence of coronary artery disease, diabetes, receipt of antihypertensive/antihyperlipidemic/antihyperglycemic treatment, diagnosed psychiatric illness, and individuals falling outside the age range of 18 to 65 years. Inclusion, exclusion criteria, and sample size determination paralleled a comparable study conducted by Cena et al. [18]. Sample size analysis was executed employing the Epi Info Sample size calculator (www.openepi.com), predicated on a metabolic syndrome prevalence rate of 52% [18], with a statistical power of 97%, a confidence interval of 95%, and a design effect of 1.0, yielding a sample size of 397 participants drawn from a pool of 2052 individuals seeking smoking cessation assistance at the clinic in 2022. Among the cohort of 397 participants, 52.4% (208 individuals) met the predefined inclusion criteria. |
| Participants | 6 | (*a*) *Cohort study*—Give the eligibility criteria, and the sources and methods of selection of participants. Describe methods of follow-up  *Case-control study*—Give the eligibility criteria, and the sources and methods of case ascertainment and control selection. Give the rationale for the choice of cases and controls  *Cross-sectional study*—Give the eligibility criteria, and the sources and methods of selection of participants | 3 | **Sampling**  This cross-sectional investigation was carried out at a smoking cessation outpatient facility within the provincial health directorate of Adana, located in Southern Turkey, targeting individuals expressing a willingness to cease smoking. Enrolment comprised all smokers seeking assistance at the smoking cessation outpatient clinic, with inclusion spanning all visitors to the facility throughout 2022 during their initial consultation. |
|  |  | (*b*) *Cohort study*—For matched studies, give matching criteria and number of exposed and unexposed  *Case-control study*—For matched studies, give matching criteria and the number of controls per case |  |  |
| Variables | 7 | Clearly define all outcomes, exposures, predictors, potential confounders, and effect modifiers. Give diagnostic criteria, if applicable | 3 | Exclusion criteria were predetermined confounding factors, including a current body mass index exceeding 30, presence of coronary artery disease, diabetes, receipt of antihypertensive/antihyperlipidemic/antihyperglycemic treatment, diagnosed psychiatric illness, and individuals falling outside the age range of 18 to 65 years. Inclusion, exclusion criteria, and sample size determination paralleled a comparable study conducted by Cena et al. [18]. |
| Data sources/ measurement | 8* | For each variable of interest, give sources of data and details of methods of assessment (measurement). Describe comparability of assessment methods if there is more than one group | *3-4* | *Blood Tests and Measurements*  *Height and weight measurements of participants were conducted during their initial visit to compute body mass index (BMI). Subsequently, biochemical parameters, including hemoglobin A1c levels, insulin, fasting blood glucose (FBG) concentrations, low-density lipoprotein (LDL), high-density lipoprotein (HDL), total cholesterol, triglyceride (TG) levels, and blood pressure (BP) readings were documented. Insulin resistance was quantified utilizing the Homeostatic Model Assessment of Insulin Resistance (HOMA-IR) formula: [FBG (mg/dL) x Fasting insulin (µU/mL)] / 405. Elevated blood pressure, in accordance with guidelines, was defined as readings surpassing 130/85 mmHg and categorized as "high" BP [19].*  *Nicotine Dependence Evaluation*  *The Fagerstrom Nicotine Dependence Test, which was used to measure the participants' nicotine dependence levels, was developed by Karl O. Fagerstrom to determine the level of physical dependence on cigarettes and consists of six questions [20].*  *This instrument comprises six closed-ended questions designed to gauge the degree of physical dependence on cigarettes, and scores on the test increase with higher levels of dependence. Individuals scoring below 5 points are classified as mild nicotine addicts, those scoring between 5 and 6 points as moderate nicotine addicts, and those scoring 7 points or above as severe nicotine addicts. The Turkish validity and reliability study of the test was conducted by Uysal et al. in 2004, revealing moderate reliability (α=0.56) [21].*  *Anxiety Assessment*  *In the study, the State-Trait Anxiety Inventory (STAI) developed by Spielberger was employed to assess the participants' anxiety levels [22]. This inventory encompasses two sections: the STAI-1, measuring state anxiety, and the STAI-2, assessing trait anxiety. The scale comprises two parts: STAI-1, which evaluates state anxiety levels, and STAI-2, which assesses trait anxiety levels. It underwent adaptation into Turkish, with validity and reliability studies conducted between 1974 and 1977 [23]. The State-Trait Anxiety Inventory consists of two separate scales with a total of twenty items each. In the State-Trait Anxiety Inventory, there are ten inverted statements. These are items 1, 2, 5, 8, 10, 11, 15, 16, 19 and 20. In the Trait Anxiety Scale, the number of reversed statements is seven. These are items 1, 6, 7, 10, 13, 16, 19. The total score of direct and reversed statements is calculated. The total score obtained for the direct statements is subtracted from the total score obtained for the reversed statements [22, 23]. According to this scale, three groups were defined for the STAI-S and STAI-T according to the literature: below 37 was considered normal, 37 to 48 was considered moderate, and above 48 was considered severe, high score [24]. Light to moderate anxiety groups were amalgamated, focusing on severe anxiety.*  *Smoking Status*  *Smoking status was classified using pack years (PY), employing three distinct categorizations 1—initially, the study's median PY of 25.0 delineated two groups: light and heavy smokers. Subsequently, in alignment with prior research, smokers were categorized into three groups based on their smoking history. 2- secondly; the first group comprised light smokers with up to 20 pack-years (PY), the second group consisted of moderate smokers with a history of 20 to 39 PY, and the third group included heavy smokers with 40 or more PY [25]. 3- thirdly; an alternative approach involved categorizing smokers into ten-year quartiles based on their smoking habits. The first (1st) quartile represented smokers with a history of up to 10 PY, the second (2nd) quartile included smokers with a history of 10 to 20 PY, the third (3rd) quartile encompassed smokers with a history of 20 to 29 years PY and the fourth (4th) quartile comprised smokers with a history of 30 years or more PY [26]. PY was calculated by multiplying the number of cigarettes smoked per day by the number of years of smoking and dividing the result by 20 [27].* |
| Bias | 9 | Describe any efforts to address potential sources of bias | 3 | Exclusion criteria were predetermined confounding factors, including a current body mass index exceeding 30, presence of coronary artery disease, diabetes, receipt of antihypertensive/antihyperlipidemic/antihyperglycemic treatment, diagnosed psychiatric illness, and individuals falling outside the age range of 18 to 65 years. Inclusion, exclusion criteria, and sample size determination paralleled a comparable study conducted by Cena et al. [18]. |
| Study size | 10 | Explain how the study size was arrived at | 3 | Sample size analysis was executed employing the Epi Info Sample size calculator (www.openepi.com), predicated on a metabolic syndrome prevalence rate of 52% [18], with a statistical power of 97%, a confidence interval of 95%, and a design effect of 1.0, yielding a sample size of 397 participants drawn from a pool of 2052 individuals seeking smoking cessation assistance at the clinic in 2022. Among the cohort of 397 participants, 52.4% (208 individuals) met the predefined inclusion criteria. |

Continued on next page

| Quantitative variables | 11 | Explain how quantitative variables were handled in the analyses. If applicable, describe which groupings were chosen and why | 3 | **Blood Tests and Measurements**  Height and weight measurements of participants were conducted during their initial visit to compute body mass index (BMI). Subsequently, biochemical parameters, including hemoglobin A1c levels, insulin, fasting blood glucose (FBG) concentrations, low-density lipoprotein (LDL), high-density lipoprotein (HDL), total cholesterol, triglyceride (TG) levels, and blood pressure (BP) readings were documented. Insulin resistance was quantified utilizing the Homeostatic Model Assessment of Insulin Resistance (HOMA-IR) formula: [FBG (mg/dL) x Fasting insulin (µU/mL)] / 405. Elevated blood pressure, in accordance with guidelines, was defined as readings surpassing 130/85 mmHg and categorized as "high" BP [19]. |
| --- | --- | --- | --- | --- |
| Statistical methods | 12 | (*a*) Describe all statistical methods, including those used to control for confounding | 4 | Metabolic syndrome parameters differed in the current population according to age, duration of smoking, time of first cigarette in the morning and level of dependence, and in previous reports, age and nicotine dependence level were predictors of metabolic syndrome parameters. Therefore, the cross-sectional design was adapted to account for these possible confounds. |
|  |  | (*b*) Describe any methods used to examine subgroups and interactions |  | Student t test, Mann Whitney U, Qi-Square and partial Spearman correlations were used in the statistical analysis. A p-value below 0.05 was deemed statistically significant. |
|  |  | (*c*) Explain how missing data were addressed |  | None applicable |
|  |  | (*d*) *Cohort study*—If applicable, explain how loss to follow-up was addressed  *Case-control study*—If applicable, explain how matching of cases and controls was addressed  *Cross-sectional study*—If applicable, describe analytical methods taking account of sampling strategy |  |  |
|  |  | (*e*) Describe any sensitivity analyses |  |  |
| Results | | | | |
| Participants | 13* | (a) Report numbers of individuals at each stage of study—eg numbers potentially eligible, examined for eligibility, confirmed eligible, included in the study, completing follow-up, and analysed |  | The data comprises 208 patients, with 97 males and 111 females. |
|  |  | (b) Give reasons for non-participation at each stage |  | Non applicable |
|  |  | (c) Consider use of a flow diagram |  | - |
| Descriptive data | 14* | (a) Give characteristics of study participants (eg demographic, clinical, social) and information on exposures and potential confounders | 5 | The average age of the entire cohort was 43.8 ±11.8 years, while the mean age upon starting smoking was 12.5 ±9.8 years, with an average smoking duration of 23.3 ± 10.7 years. Basal glucose levels averaged 96.0 ±18.6 mg/dL. STAI-A and STAI-T Anxiety scores were recorded as 49.4 ± 6.6 and 50.0 ± 7.0, respectively. When categorizing smoking status into heavy and light based on median values, 53.7% of participants were identified as heavy smokers. Prevalence rates of meeting Metabolic Syndrome criteria were 41.8% for TG, 33.7% for HDL, 23.6% for BP, and 16.4% for FG.  Table 1 outlines the characteristics of the sample and compares values between genders. As anticipated, females exhibited significantly lower HDL values (p< 0.001), higher SBP values (p< 0.001), and lower DBP values (p< 0.001). The proportion of heavy smokers (p= 0.008), individuals with triglyceride levels meeting metabolic syndrome criteria (p= 0.001), and those with high blood pressure (p= 0.006) were significantly higher among males. |
|  |  | (b) Indicate number of participants with missing data for each variable of interest |  |  |
|  |  | (c) *Cohort study*—Summarise follow-up time (eg, average and total amount) |  |  |
| Outcome data | 15* | *Cohort study*—Report numbers of outcome events or summary measures over time |  |  |
|  |  | *Case-control study—*Report numbers in each exposure category, or summary measures of exposure |  |  |
|  |  | *Cross-sectional study—*Report numbers of outcome events or summary measures |  |  |
| Main results | 16 | (*a*) Give unadjusted estimates and, if applicable, confounder-adjusted estimates and their precision (eg, 95% confidence interval). Make clear which confounders were adjusted for and why they were included |  |  |
|  |  | (*b*) Report category boundaries when continuous variables were categorized | 5 | *In Table 1, heavy smoking was represented by the median, with all subjects having smoked for a median duration of 25.0 years. Table 2 categorized values based on light, moderate, and heavy smoking levels by pack-years, further divided into quartiles of 10 years each.* |
|  |  | (*c*) If relevant, consider translating estimates of relative risk into absolute risk for a meaningful time period |  |  |

Continued on next page

| Other analyses | 17 | Report other analyses done—eg analyses of subgroups and interactions, and sensitivity analyses |  |  |
| --- | --- | --- | --- | --- |
| Discussion | | | | |
| Key results | 18 | Summarise key results with reference to study objectives | 6-9 | The etiology of metabolic syndrome remains subject to debate, yet smoking emerges as a notable modifiable risk factor. Extensive research indicates that smoking is linked to lipid irregularities, endothelial dysfunction, and a prothrombotic state, all of which constitute components of metabolic syndrome [28, 29]. This association is further underscored by evidence suggesting that smoking may exacerbate insulin resistance, contributing to metabolic and hemodynamic aberrations [29]. While certain investigations have demonstrated a positive correlation between smoking and the prevalence of metabolic syndrome [30, 31], conflicting findings have been reported in other studies [32, 33]. Notably, a study among Turkish women suggested a reduced risk of metabolic syndrome among smokers [34].  In exploring the relationship between anxiety and metabolic syndrome, evidence suggests a tenuous link [35, 36]. A recent meta-analysis of 18 cross-sectional studies examining anxiety's impact on metabolic syndrome risk reported a modest yet discernible increase in risk (OR=1.07) [37]. Furthermore, a registry review revealed elevated cardiometabolic risks among individuals with anxiety, encompassing conditions such as diabetes, hypertension, hyperlipidemia, and obesity [38]. Given the profound influence of lifestyle on metabolic syndrome and the generally unhealthy lifestyles prevalent among psychiatric patients, it is posited that poor lifestyle choices may contribute to the observed association between anxiety disorders and metabolic syndrome [39].  In the United States, research indicates that individuals with a history of smoking are more predisposed to metabolic syndrome compared to non-smokers, constituting a significant risk across genders and age groups [40, 41]. Cigarette smoking emerges as a notable behavioral contributor to metabolic syndrome and is linked to a spectrum of diseases [18, 42]. While prior investigations have explored this association using metrics like daily tobacco consumption or total years of smoking. We compare genders using a median-based estimate over pack-years, employing a three-tier assessment (light, moderate, heavy) akin to previous research, as well as quartiles. Our findings underscore the dose-dependent relationships between smoking, metabolic syndrome criteria, and levels of addiction.  Moreover, our study illuminates the concordance between crucial metabolic parameters and anxiety scores within our cohort. While previous studies have typically focused on daily tobacco consumption or cumulative smoking duration, our study offers a nuanced analysis incorporating gender-specific median values, pack-years, a three-tier assessment (light, moderate, heavy), and quartiles, aligning with prior research [18, 42, 43]. Smoking represents a significant behavioral determinant of metabolic syndrome and is intricately linked with various syndrome-associated conditions. Our data accentuate the dose-dependent associations of smoking behaviors and the interplay between addiction levels and metabolic syndrome criteria. Additionally, our study unveils, for the first time, parallels between key metabolic components and anxiety scores within a cohort of 208 smokers.  Previous studies, such as that by Wada et al., have underscored the existence of a dose-response relationship between metabolic syndrome (MS) and smoking, implicating smoking as a contributing factor to this syndrome [42]. Additionally, Cena et al. have noted that the effects previously attributed to insulin resistance persist even when considering smoking effects independently of insulin resistance [18]. Notably, nicotine, carbon monoxide, and other smoking metabolites have been implicated in inducing insulin resistance [44]. Investigations, such as those conducted as part of The Korea National Health and Nutrition Examination Survey (KNHANES), have revealed a correlation between increased MS components and the level of tobacco consumption, suggesting a dose-dependent relationship [26]. Furthermore, this compromised metabolic profile may persist even during the early stages of smoking cessation, improving gradually with continued cessation. Studies have reported reduced risks of MS and diabetes in individuals who quit smoking, with risk reduction extending up to 20 years after cessation in some instances [42-45]. Our findings align with existing literature, indicating that impaired lipid and insulin profiles likely endure and contribute to MS among former heavy smokers even after smoking cessation, thereby highlighting a deteriorating metabolic state in heavy smokers with prolonged exposure.  The absence of dose-dependent worsening in insulin and HOMA-IR levels in our study compared to the existing literature is a notable and distinctive finding. Despite this, we observed that criteria for metabolic syndrome and important metabolic components were affected at various levels, including SBP, DBP, HgbA1C, and LDL. Consequently, mechanisms other than insulin resistance can adversely influence metabolic syndrome parameters, aligning with a dose-response curve, revealing an ongoing deterioration independent of insulin resistance, just as suggested by Cena et al [18].  A comparative analysis of men and women revealed no significant differences in BMI and age. However, as anticipated, disparities were observed in HDL values, with men exhibiting higher SBP and DBP levels and a higher prevalence of poor triglyceride profiles. Another notable difference was the higher proportion of heavy drinkers among men; this may be evidence of a clear relationship between the drinking profile and SBP, DBP and triglyceride profiles. This observation is supported by our study sample, which adjusts for confounding variables such as BMI, age, metabolic syndrome-related diseases, or psychiatric illness status.  Previous research by Chen et al. found no significant relationship between fasting blood glucose levels and tobacco consumption. At the same time, conflicting evidence was reported by Will et al., who suggested an association between increasing tobacco consumption and the incidence of type 2 diabetes [31, 45]. These contradictory findings regarding the effect of smoking on fasting blood glucose levels underscore the complexity of this relationship. Thus, while our study did not reveal a distinct difference in fasting blood glucose, definitive conclusions cannot be drawn.  Our study also evidently impacted LDL profile, an important risk factor for coronary artery disease, although LDL is not among the criteria for metabolic syndrome. Specifically, LDL profile was lower in light smokers or those with less than 10 years of smoking history. However, the duration of this effect could not be determined statistically in our study. While triglyceride levels increased significantly in accordance with the median smoking duration (25 years), the duration of this effect remained unclear statistically like LDL profile. This ambiguity may be attributed to the cross-sectional nature of our study design. As anticipated, SBP and DBP profiles were significantly impaired in heavy smokers, consistent with findings in the literature [18]. Interestingly, anxiety values did not exhibit significant differences based on smoking status. Although there was a tendency for anxiety to increase, this trend was not substantial enough to infer a definitive parallelism between smoking status and anxiety. However, it is noteworthy that trait anxiety scores were notably elevated among heavy smokers.  Our study adds to the existing literature by examining the relationship between anxiety scores and metabolic syndrome components. Consistent with previous research, our findings suggest that SBP and DBP values are influenced by the anxiety levels of patients, as discussed elsewhere in the literature [46, 47]. Additionally, we observed a parallel worsening of nearly all metabolic syndrome criteria with high trait anxiety scores.  Prior studies have indicated elevated anxiety scores in patients with polycystic ovary syndrome. Furthermore, it has been established in the literature that a poor metabolic profile can lead to anxiety [35, 48, 49]. Given these findings, it is evident that individuals seeking smoking cessation, particularly those with a compromised metabolic profile, may need integrated services addressing both physical and mental health concerns, including anxiety. |
| Limitations | 19 | Discuss limitations of the study, taking into account sources of potential bias or imprecision. Discuss both direction and magnitude of any potential bias | 8-9 | Our study has both strengths and limitations. One limitation is the absence of waist circumference measurement and information on the prevalence of metabolic syndrome, which could provide further insights into the relationship between smoking, anxiety, and metabolic health. Additionally, the study's cross-sectional design may introduce bias in responses, particularly regarding pack/year estimation.  However, our study benefits from several strengths. We included height/weight measurements and applied strict exclusion criteria, allowing for a more accurate assessment of the effects of smoking on metabolic health. Furthermore, our study focused on individuals attending a smoking cessation clinic, providing valuable insights into the interplay between smoking, anxiety, addiction levels, and metabolic syndrome components in this population.  Despite these limitations, our study aligns with existing literature and offers new insights, highlighting the potential benefits of smoking cessation in preventing metabolic abnormalities. |
| Interpretation | 20 | Give a cautious overall interpretation of results considering objectives, limitations, multiplicity of analyses, results from similar studies, and other relevant evidence |  |  |
| Generalisability | 21 | Discuss the generalisability (external validity) of the study results | 8 | Our study benefits from several strengths. We included height/weight measurements and applied strict exclusion criteria, allowing for a more accurate assessment of the effects of smoking on metabolic health. Furthermore, our study focused on individuals attending a smoking cessation clinic, providing valuable insights into the interplay between smoking, anxiety, addiction levels, and metabolic syndrome components in this population. Despite some limitations, our study aligns with existing literature and offers new insights, highlighting the potential benefits of smoking cessation in preventing metabolic abnormalities |
| Other information | |  | | |
| Funding | 22 | Give the source of funding and the role of the funders for the present study and, if applicable, for the original study on which the present article is based |  | none |

*Give information separately for cases and controls in case-control studies and, if applicable, for exposed and unexposed groups in cohort and cross-sectional studies.

**Note:** An Explanation and Elaboration article discusses each checklist item and gives methodological background and published examples of transparent reporting. The STROBE checklist is best used in conjunction with this article (freely available on the Web sites of PLoS Medicine at http://www.plosmedicine.org/, Annals of Internal Medicine at http://www.annals.org/, and Epidemiology at http://www.epidem.com/). Information on the STROBE Initiative is available at www.strobe-statement.org.
